# EEG-based classification of epilepsy and PNES: EEG microstate and functional brain network features

**DOI:** 10.1186/s40708-020-00107-z

**Published:** 2020-05-29

**Authors:** Negar Ahmadi, Yulong Pei, Evelien Carrette, Albert P. Aldenkamp, Mykola Pechenizkiy

**Affiliations:** 1 Department of Mathematics and Computer Science, Eindhoven University of Technology, TU/e, P.O.Box: 513, 5600MB Eindhoven, NL The Netherlands; 2 Neurology Department, Ghent University Hospital, Ghent, Belgium; 3 Department of Electrical Engineering, Eindhoven University of Technology, Eindhoven, The Netherlands

**Keywords:** EEG microstate, Functional network, Classification, Epilepsy, PNES

## Abstract

Epilepsy and psychogenic non-epileptic seizures (PNES) often show over-lap in symptoms, especially at an early disease stage. During a PNES, the electrical activity of the brain remains normal but in case of an epileptic seizure the brain will show epileptiform discharges on the electroencephalogram (EEG). In many cases an accurate diagnosis can only be achieved after a long-term video monitoring combined with EEG recording which is quite expensive and time-consuming. In this paper using short-term EEG data, the classification of epilepsy and PNES subjects is analyzed based on signal, functional network and EEG microstate features. Our results showed that the beta-band is the most useful EEG frequency sub-band as it performs best for classifying subjects. Also the results depicted that when the coverage feature of the EEG microstate analysis is calculated in beta-band, the classification shows fairly high accuracy and precision. Hence, the beta-band and the coverage are the most important features for classification of epilepsy and PNES patients.

## Introduction

Abnormal electrical activity in the brain can cause epileptic seizures. When a person has repeated seizures, this condition is called epilepsy. Hence epilepsy is a transient occurrence of signs and/or symptoms due to abnormal excessive and/or synchronous neuronal activity in the brain [1]. The visible effect (i.e., the seizure) varies from temporary confusion, loss of awareness. Patients seldomly are prior aware of the occurrence of seizures increasing the risk of physical injury. Psychogenic nonepileptic seizures (PNES) are events resembling an epileptic seizure, but without the characteristic electrical discharges associated with epileptic seizures [2] that have a psychogenic origin [3]. The symptoms of PNES usually reflect a psychological conflict that is often associated with distress, disability, and have a poor prognosis when not timely and accurately diagnosed and treated [4]. PNES episodes are not purposely produced by the patient, and the patient is not aware that the seizures are non-epileptic, so the patient may become anxious when having these symptoms. The presentation of the differential diagnosis should be done early in the course of treatment for better patient acceptance, and treatment options should be presented early in the evaluation period [5].

Early diagnosis of epilepsy or PNES is critical. Because of delay in early diagnosis, many patients experience significant morbidity from inappropriate treatment, including adverse effects of antiepileptic drugs and aggressive interventions, such as intubation for pseudostatus epilepticus [6]. However, PNES may be misdiagnosed as epilepsy, and patients are often treated with an incorrect diagnosis [7] with potentially important side-effects. The failure to recognize the psychological cause of the disorder detracts physicians from addressing associated psychopathology, and enhances secondary somatization processes [5].

During a PNES, the brain’s electrical activity remains normal but in case of an epileptic seizure, interictal epileptiform discharge (IED) occurs. Hence optimal differential diagnosis between epilepsy and PNES can be made based on video-EEG monitoring, during which an attempt is done to record a seizure while recording video and EEG. Besides interpretation of the semiology on the video, the EEG can help in the differentiation between both. If muscle activity is not to prominent, the occurrence of ictal electrical discharges during a seizure can confirm the diagnosis of epilepsy over PNES. When no ictal discharges are observed not certain diagnosis can be made; however, semiology often helps in the diagnosis. In Ref. [8] a clear guidance on standards for the diagnosis of PNES has been delineated. However, long-time EEG monitoring and recording are quite expensive. If we can exclude PNES patients based on a short-term EEG recording, this would reduce the recording cost and burden waiting lists for EEG monitoring units.

It has been shown that the evolutionary pattern of the frequencies of rhythmic movement artifacts on EEG during PNES differs from that of epileptic seizure [9]. Convulsive PNES were demonstrated to display a characteristic pattern of rhythmic movement artifact that remains stable over time during the event, whereas the EEG activity during convulsive epileptic seizure tends to evolve over time [9]. This finding indicated that time–frequency analysis of data from a wristband movement monitor has the potential to be utilized as a diagnostic tool to differentiate between PNES and epileptic seizure with a high sensitivity and specificity [10, 11]. Using a seizure detection and classification algorithm, Naganur et al. [11] examined the diagnostic utility of an automated analysis with an ambulatory accelerometer using EEG moments that show seizure-like activity. Also, in our previous work [12] we classified the PNES and epileptic seizure with a very high accuracy using EEG data including seizure-like activities.

However, as we mentioned earlier, the issue in EEG/video monitoring of the patients with epileptic seizure is that the IED occurs unpredictably. Hence, it is necessary to record EEG for a very long time to see if any epileptiform discharges occur and then use those data for further analysis. Therefore, the aim of this research is finding discriminative features in short-term EEG signals and brain networks in epilepsy patients compared to PNES subjects in the absence of an IED (or seizure) to effectively classify these two groups. Classification of the disorders using IED-free EEG data makes the classification quite challenging. To the best knowledge of the authors, no similar work has been reported in the literature.

At first, we use EEG signal features for automatic classification of the groups. The first EEG signal analysis step is known as feature extraction that aims at describing the EEG signals by (ideally) a few relevant values called features [13]. Such features should capture the information embedded in EEG signals that is relevant to describe the mental states to identify, while rejecting the noise and other non-relevant information. Hence, the purpose of feature extraction is not only to reduce the dimensionality but also to extract more useful/dominant information hidden in the signals by avoiding unnecessary or redundant information.

We also apply the functional brain network analysis to extract network features for classification purpose. A functional network is a mathematical representation of the brain and is defined by a collection of nodes and links between pairs of nodes. Nodes in a functional brain network represent brain regions, while links represent functional connections corresponding to the magnitude of the temporal correlation between node pairs [14]. Functional connectivity is highly time-dependent, often changing in a matter of tens or hundreds of milliseconds as functional connections are continually modulated by sensory stimuli and task context. A network formulation simplifies the analysis of brain by providing mathematical tools able to capture different aspects of its organization in a compact and straightforward manner [15, 16]. Graph theoretical methods have been extensively applied to many neuroimaging datasets to describe the topological properties of both functional and structural networks.

In the absence of an IED, the EEG signals of epilepsy and PNES are quite similar and common EEG signal and/or network features may not act as accurate discriminative parameters for classification purpose. Hence, we need to apply a capable analysis with high resolution in time to extract discriminative features. Hence, we also apply EEG microstate analysis to explore if abnormalities in microstates can identify patients with epilepsy and PNES with high accuracy. Microstate analysis is an alternative EEG representation that defines states of the multichannel EEG recording by spatial topographies of electric potentials over the electrode array. This method was first proposed by Lehmann et al. [17], who showed that the alpha frequency band (8–12 Hz) of a multichannel resting-state EEG recording can be parsed into a few number of discrete quasi-stable states that remain dominant for around 80–120 µs before abruptly transitioning to another state. These quasi-stable states are defined by topographic maps of electric potentials recorded in a multichannel array over the scalp. These periods of states are called functional microstates and the discrete spatial configurations are known as microstate classes/maps.

Compared to other EEG analysis techniques, spatial analysis of EEG using microstates has several advantages. Most importantly, the spatial topography of the EEG recording can be defined at any data point independently of the preceding topography and therefore has millisecond resolution. Hence, microstates are better suited to detect rapid, dynamic activity in large-scale neurocognitive networks than many traditional methods like frequency power EEG analysis [18]. The spatial EEG signal analysis with microstates simultaneously considers the signal from all electrodes to create a global representation of a functional state. The rich syntax of the microstate time series offers a variety of new quantifications of the EEG signal with potential neurophysiological relevance [19]. In addition, parsing the EEG into microstates can be used to select epochs of interest that correspond to a certain microstate class, which can be further examined using other analysis methods such as time–frequency analysis. Therefore, EEG microstate analysis offers a capable, cost-worthy and clinically translatable neurophysiological approach to study large-scale neural networks and investigate temporally coherent network activity, as it has been suggested to reflect global functional states of the brain in health and brain disorders [19–22].

The rest of this paper is structured as follows. In Sect. 2, the mathematical methods that we apply for classification purpose are presented. Classification results are presented in Sect. 3, following by concluding remarks in Sect. 4.

## Materials and methods

### Clinical EEG data

The dataset used in this section was obtained from Ghent University Hospital in Belgium with whom a larger multidisciplinary brain research program, called Neu3CA [23], is ongoing. The EEG recordings were obtained from 5 epilepsy and 5 PNES patients. The recordings from each patient include 27 EEG recording electrodes (based on the standard 10–20 acquisition system) and reference (G2) on the right mastoid bone plus the ground (G1) on the left mastoid bone. The sampling rate of all data channels is 256 Hz and the duration of each acquired raw EEG data is 3 h. The 27 channels are Fp1, Fpz, Fp2, F7, F3, Fz, F4, F8, C3, Cz, C4, T7, T8, P7, P8, P3, Pz, P4, O1, Oz, O2, T9, T10, FT9, FT10, TP9 and TP10.

For each patients groups, 50 IED-free epochs, which are termed as subjects, with the duration of 16 s and with the same classification labels were extracted as they contain the least amount of noise or artifact. Thus, we have 100 subjects including 50 Epilepsy and 50 PNES epoches. Then, all epochs were band-passed filtered for the frequency range of 1–40 Hz to further minimize contamination by high-frequency artifact. Finally, each segment is decomposed to its sub-band frequencies. The main frequency sub-bands are delta (below 4 Hz), theta (4–8 Hz), alpha (9–13 Hz), beta (14–30 Hz) and gamma (above 30 Hz).

To avoid overfitting, we conduct classification experiments using cross-validation. For this purpose, we randomly select 1 Epilepsy subject and 1 PNES subject where each subject includes 10 epoches with the same label. Therefore, there are totally $5\times 5=25$ pairs of cross-validation experiments. The results reported in this paper will be the average of these 25 pairs.

### EEG signal analysis

In this paper, a wavelet-based time–frequency scheme [23] is applied to decompose the EEG signals into its sub-bands. The wavelet decomposition is a smooth and quickly vanishing oscillating function with good localization in both frequency and time. Then we use different features based on the EEG signals to transform raw signals in each sub-bands into more informative signatures or fingerprints of the brain network. Note that the signal features are extracted from each single EEG channel and then all of the extracted features are used as the input data for the classifiers. Here, the selected signal features are presented below briefly.

#### Energy

Discrete time signals are the signals that can be defined and represented at certain time instants of the sequence. As we mentioned before, the sampling rate of all data channels is 256 Hz. It means that the voltage of brain at different locations has been recorded every 1/256 s. Hence, the EEG signals can be considered as discrete signals. In the discrete domain, the energy of the signal is given by [24]

1 $$ E = \sum\limits_{{i = 1}}^{n} {x_{i}^{2} }  $$

where *i* represents the recording time instant, $x_i$ the voltage of signal at *i* and *n* the total number of time instants.

#### Entropy-based features

Entropy measure shows the amount of randomness and uncertainty in the signal; therefore, the more fluctuating signal has a higher value of entropy. In other words, entropy reflects how well one can predict the behavior of each respective part of the trajectory from the other. Basically, higher entropy indicates more complex or chaotic systems, thus, less predictability.

*Shannon entropy (ShE)*: Shannon entropy [25] is a non-linear measure quantifying the degree of complexity in a signal. Let *X* be a set of a discrete EEG signal variables $X=\{x_1,x_2,\ldots ,x_n\}; x_i\in R^d$. Now, the Shannon entropy is defined as

2 $$\begin{aligned} \text {ShE}=-\sum _{i=1}^{n}p(x_i)\ln p(x_i) \end{aligned}$$

where $p(x_i)$ is probability of $x_i\in X$ satisfying $\sum _{i=1}^{n}p(x_i)=1$.

*Spectral entropy (SE)*: Spectral entropy (SE) computation uses Shannon’s entropy formula to represent the power spectral densities of the EEG signal as probabilities [26]. For this purpose, fast Fourier’s transformation (FFT) is used to obtain the spectrum. The normalized SE corresponding to the frequency range $[f_1,f_2]$ is calculated from 1-s epochs of 27-channel EEG signals of epileptic and PNES group as follows [27]:

3 $$\begin{aligned} \text {SE}[f_1,f_2]=\frac{\sum _{f_i=f_1}^{f_2}P_n(f_i)\log (P_n(f_i))}{\log (N[f_1,f_2])} \end{aligned}$$

where $N[f_1,f_2]$ equals the total number of frequency components in the frequency range and $P(f_i)$ represents the probability of the *i*th frequency component. Each 1-s, 27-channel EEG data epoch (27 channels $\times$ 256 instants/s) is represented by a 27-component SE vector (27 $\times$ 1), called SE feature vectors.

*Renyi entropy (RE)*: Renyi entropy, as an index of diversity, is generalizations of Shannon entropy that depend on a parameter [28]. If $p(x_i)$ is a probability distribution on a finite set, its Renyi entropy of order $\alpha$ is defined as $\text {RE}=\frac{1}{1-\alpha }\ln \sum _{i=1}^{n}p(x_i)^{\alpha }$, where $0<\alpha <\infty$. Renyi entropy approaches Shannon entropy as $\alpha \rightarrow 1$ [29]. In our study, the value of $\alpha$ is taken as 2. Steps involved in RE are quite similar to computing ShE.

#### Fractal dimension-based features

Fractals are mathematical sets with a high degree of geometrical complexity that can model many natural phenomena. A very important characteristic of fractals, useful for their description and classification, is their fractal dimension. The fractal dimension of a set in metric space, such as an EEG signal, can be computed from several different measures [30].

*Fractal box dimension (FBD)*: For calculating this measure, a box with different side lengths is used to describe the change of the signal waveform. Smaller side lengths of the box lead to a longer calculation time, but the recognition rate of the signal will increase. Smaller side lengths of the box lead to a longer calculation time, but the recognition rate of the signal will increase. The idea is to apply continuous hypercube mesh coverage to the curve. If we consider *X* as a non-empty compact subset of the real plane, then the capacity dimension is defined as

4 $$\begin{aligned} \text {FBD}=\lim _{\epsilon \rightarrow 0} \frac{\log N_{\text{ min }}(\epsilon )}{\log (1/\epsilon )} \end{aligned}$$

where $N_{\text{ min }}(\epsilon )$ is the smallest number of boxes with a side length $\epsilon$ required to cover *X*. The box dimension merely represents the geometric dimension of the signal, but does not reflect the density distribution in the planar space.

*Higuchi fractal dimension (HFD)*: The HFD is a fast non-linear computational method for obtaining the fractal dimension of signals even when very few data points are available [31]. HFD is used to quantify the complexity and self-similarity of a signal. To compute the HFD, the dataset is divided into a *k*-length sub-dataset as $x_k^m:x_m,x_{m+k},x_{m+2k},...,x_{m+(\frac{n-m}{k})k}$, where *n* is the total length of the data sequence, *k* is a constant and $m=1,2, ..., k$. The length $L_m(k)$ for each sub-dataset is then computed as

5 $$\begin{aligned} L_m(k)=\frac{\sum _{i=1}^{N-\frac{m}{k}} \mid x_{m+ik}-x_{m+(i-1)k} \mid (n-1) }{(\frac{n-m}{k})k} \end{aligned}$$

where the mean of $L_m(k)$ for each *k* is computed to find the HFD as

6 $$\begin{aligned} \text {HFD}=\frac{1}{k}\sum _{m=1}^{k}L_m(k) .\end{aligned}$$

It should be mentioned that to determine the maximum value for *k*, we followed the recommendation of Doyle et al. at [32]. For this purpose, a maximum number of reconstructed datasets, e.g., $K_{\text {max}}$=5, is determined by the user. For each reconstructed dataset the curve length is calculated and plotted against its corresponding *k* value on a log-log scale. The resulting slope, fitted by a least-squares method, represents the fractal dimension of the original data. Determining $K_{\text {max}}$ is by a process of examining the data and plotting the fractal dimension over a range of $K_{\text {max}}$; the point at which the fractal dimension plateaus is considered a saturation point beyond which no benefit could be gained from further calculations. Best results for the current data were obtained using a $K_{\text {max}}$=20.

*Katz fractal dimension (KFD)*: The KFD is derived directly from the waveform, eliminating the pre-processing step of creating a binary sequence, can be defined as [33]

7 $$\begin{aligned} \text {KFD}=\frac{\log _{10}(n)}{\log _{10}(\frac{d}{L})+\log _{10}(n)} \end{aligned}$$

where *n* is the number of steps in the curve, *L* is the total length of the signal that is to say, the sum of the distance between successive points. Also *d* is the Euclidean distance between the first point in the series and the point that provides the furthest distance with respect to the first point.

### Functional network analysis

Various complex network measures can be used to analyze the functional brain network and characterize one or more aspects of local or global brain connectivity. To create a functional network, a matrix containing the EEG channels pairwise correlations is required. Thus, one needs to calculate the synchronizations among all pairs of signals and deduce the respective correlation (or adjacency) matrix. Applying a synchronization measure results in the calculation of a correlation matrix with each row representing a node and each column on that row representing the relationship between the current node and every other node in the network. Links between nodes are weighted which represent strength of correlation or causal interactions in functional networks.

In this paper, a synchronization measure based on the horizontal visibility graph (HVG) is applied to calculate correlation matrix and construct the functional network. Visibility algorithms are a family of methods that map signals as graphs nonlinearly  [34–36]. The HVG algorithm provides an effective method to map EEG signals to a graph permitting a mutual relationship between dynamical properties of signals and topological properties of the graph. Therefore, the information on EEG signals is obtained just by analyzing the characteristics of the graph. In our previous works, we showed that the synchronization measure based on the HVG algorithm is a robust measure for finding correlation among chaotic, noisy and stochastic signals [37], and also this measure is less sensitive to the brain volume conduction effect and is able to predict the coupling degree correctly even with strongly overlapping signals [38]. This synchronization measure is presented here shortly.

#### HVG-based synchronization measure

**Fig. 1:**
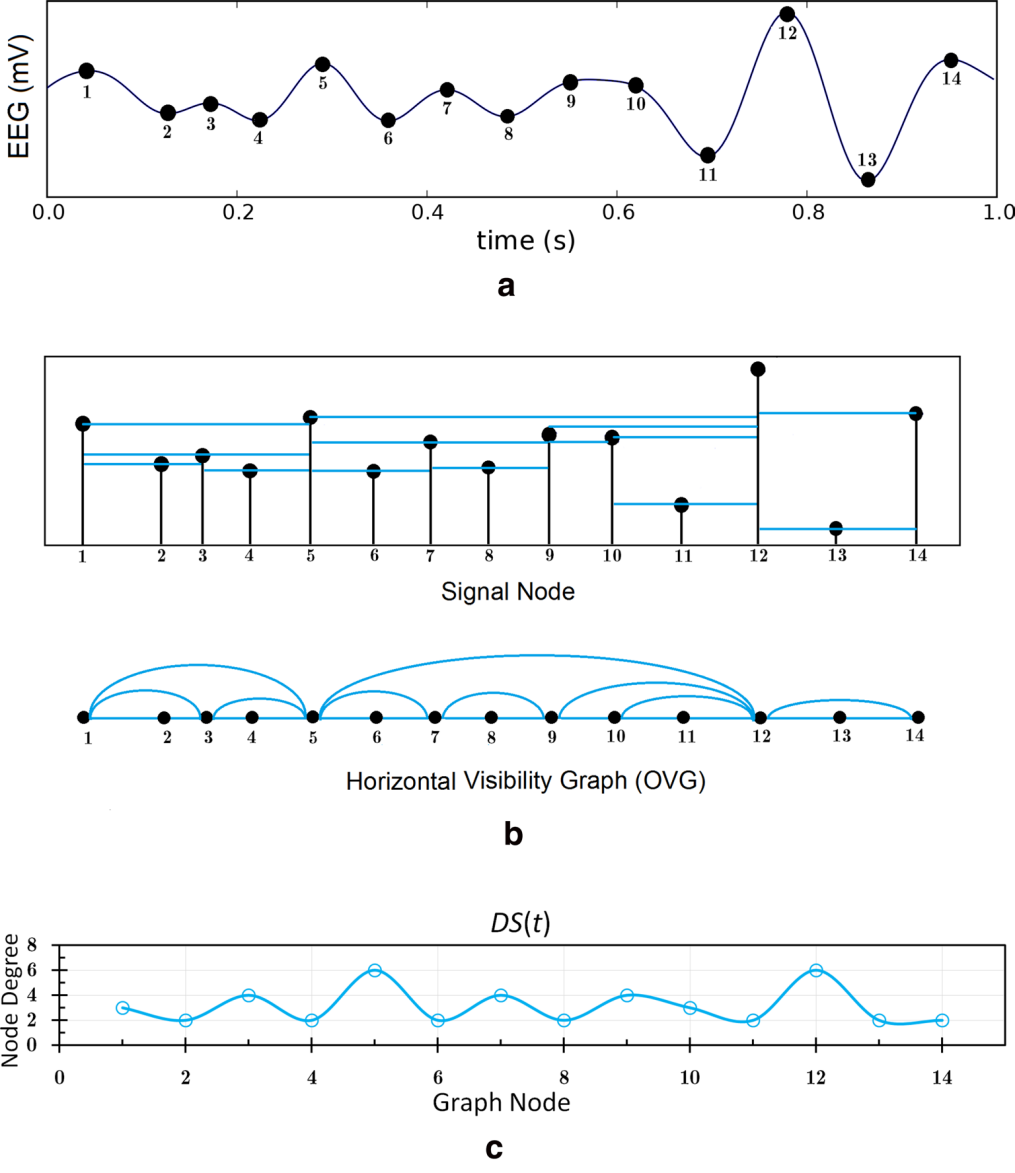
**a** An EEG time series (filled circles represent time points), **b** top: applying HVG criteria on time points, bottom: corresponding graph, and **c** corresponding degree sequences of the HVG for such time points

Let *x*(*t*) be a univariate time series of *N* discrete data ($t=1,2,...,N$). The visibility graph algorithm converts the time series *x*(*t*) to a graph, as a data point *x*(*t*) is mapped into a node in the graph. The time point (i.e., a point on the time series) represents a moment in which the data are recorded (see Fig. 1a). By applying the HVG algorithm, an EEG time series of size *N* maps to a visibility graph with *N* nodes. In this algorithm, two arbitrary data nodes $t^{*}$ and $t^{\star }$ in the graph are connected if [35]

8 $$\begin{aligned} x(t^{*})>x(t)~\text {and}~x(t^{\star })>x(t)~~\text {for all { t} such that:}~(t^{*}< t < t^{\star }). \end{aligned}$$

According to the HVG geometric criterion, two data points are connected if one can draw a horizontal line in the time series joining them that does not intersect any intermediate data height. Therefore, by applying the HVG, a signal of size *N* maps to a graph with *N* nodes, as the first node in Fig. 1b is associated with the first time point in Fig. 1a. The second node corresponds to the second time point of the EEG time series, and so on.

After constructing the visibility graph, the degree of each node is determined. The degree of node *t* is the number of links connected to node *t*. Therefore, by counting the number of links that have node *t* as an endpoint, we can determine the degree of each node. Then, by considering the degrees of all nodes, a degree sequence (DS) time series is obtained. The corresponding DSs of the HVG algorithms are shown in Fig. 1c as time series. Next, the similarity of two time series *x*(*t*) and *y*(*t*) is approximated by calculating the *Cross-Correlation* (CC) function between the DSs of the corresponding visibility graphs. The cross-correlation function measures the linear correlation between two time series as a function of their delay time, which is of interest because such a time delay may reflect a causal relationship between the time series. The CC between two time series *x*(*t*) and *y*(*t*) with the same *N* samples’ length, where *t* denotes discrete time $(t=1,...,N)$, is expressed as

9 $$\begin{aligned} \text {CC}=C_{xy}(h)=\frac{1}{N-h}\sum _{t=1}^{N-h}x(t+h)y(t), \end{aligned}$$

where $t=1,...,N$ denotes discrete time and $h\in \{-(N-1),..., 0,..., N-1\}$ denotes time lag. Here, CC = $\pm 1$ presents the complete linear direct and inverse correlations, respectively, and CC = 0 indicates lack of linear correlation for a given time lag.

After constructing the functional network at each frequency sub-band, some selected complex network measures are determined as following to detect aspects of the brain network.

#### Clustering coefficient

The clustering coefficient assesses the degree to which nodes tend to cluster together. In brain network studies, the clustering coefficient is considered to be a measure of the local connectivity of the functional brain network. Brain networks are “small worlds” in which different functional units can work independently but are connected to each other through hubs. A high clustering coefficient indicates the presence of local cliques forming specialized functional units. Given a weighted network *G*, the local clustering coefficient $c_i$ for node *i* is defined as [39]

10 $$\begin{aligned} c_i=\frac{2}{d_i(d_i-1)}\sum _{i,k}(\tilde{w}_{ij}\cdot \tilde{w}_{jk}\cdot \tilde{w}_{ki})^{1/3}, \end{aligned}$$

where $\tilde{w}_{ij}=w_{ij}/{\text{max}}(w_{ij})$ is the scaled weight. Here, $d_i(d_i-1)/2$ is the maximum possible number of links when the subgraph of neighbors of node *i* is completely connected. The global clustering coefficient for the whole graph is the average of the local values and is defined as  [40]

11 $$\begin{aligned} C=\frac{1}{N}\sum _{i}^{N}c_i, \end{aligned}$$

where *N* is the number of nodes in the graph. It is clear that $0\le c_i\le 1$ and $0\le C\le 1$. Note that $c_i=1$ if node *i* is the center of a fully interconnected cluster and $c_i=0$ if the neighbors of node *i* are not connected to each other.

#### Strength

Strength is one of the most basic structural properties of a weighted graph. The vertex strength is defined as the sum of weights of links connected to the vertex and is formalized as

12 $$\begin{aligned} S_i=\Sigma w_{ij}. \end{aligned}$$

where $j\in neighbor(i)$ and *w* represents the weighted adjacency matrix, in which $w_{ij}$ is the weight on the edge between node *i* and *j* [41].

#### Betweenness centrality

Centrality refers to the relative importance of a vertex within the network. Mostly, the vertices in a network with higher centrality index values are perceived as being the more important vertices. Betweenness centrality quantifies the number of times that a node acts as a bridge along the shortest path between two other nodes. In an undirected network, a path between two nodes that has the minimum number of links is referred to as the shortest path between these two nodes. In the context of brain network analysis, a brain region (or EEG recording site) has a high betweenness centrality index if it is strategically located as a midpoint between several pairs of brain regions, and therefore, controls the flow of information across the brain network.

Consider an undirected graph $G=(V,E)$, where *V* and *E* denote its node and link set, respectively. For three distinct nodes $v_1,v_2,v_3\in V$, let $\sigma _{v_1,v_3}\ne 0$ be the number of shortest paths between $v_1$ and $v_3$ in *G*, and let $\sigma _{v_1,v_3}(v_2)$ be the number of shortest paths between $v_1$ and $v_3$ that pass through $v_2$. The betweenness centrality index of node $v_2$ is defined as [42]

13 $$\begin{aligned} B(v_2)=\sum _{v_1\ne v_2\ne v_3\in V}\frac{\sigma _{v_1,v_3}(v_2)}{\sigma _{v_1,v_3}}. \end{aligned}$$

The average node betweenness centrality of the graph is defined as follows:

14 $$\begin{aligned} \bar{B}(G)=\frac{1}{N}\sum _{v_2\in V}B(v_2). \end{aligned}$$

The betweenness centrality lies between zero and $$\left( {\begin{array}{c}N-1\\ 2\end{array}}\right) $$, where the value 0 is obtained if and only if all neighbors of $v_i$ induce a maximal clique in *G*.

#### Eigenvector centrality and largest eigenvalue

Eigenvector centrality is a global measure of centrality, as it does not focus on the immediate vicinity of nodes but instead considers all possible indirect connections. It operates under the premise that connections to nodes that are themselves well-connected should be given more weight than connections to less well-connected nodes. Eigenvector centrality for all nodes in the network, then, is simply given by the eigenvector corresponding to the largest eigenvalue (also called the Perron eigenvalue). In brain network studies, the eigenvector centrality is a measure that approximates the centrality or the importance of a brain region to the corresponding functional network. Eigenvector centrality attributes a value to each voxel in the brain, such that a voxel receives a large value if it is strongly correlated with many other nodes that are themselves central within the network. A brain region has higher eigenvector centrality if its neighbors are also highly central. It has been demonstrated that eigenvector centrality is a computationally efficient tool for capturing intrinsic neural architecture on a voxel-wise level [43].

For a matrix $\mathbf {A} \in R^{N\times N}$, a number $\lambda$ is an eigenvalue if, for some vector $\vec {c}\ne 0$ [41],

15 $$\begin{aligned} \mathbf {A}\vec {c}=\lambda \vec {c}. \end{aligned}$$

Here, the centrality vector $\vec {c}$ is the eigenvector of the adjacency matrix $\mathbf {A}$ associated with the eigenvalue $\lambda$. In general, eigenvectors give the direction of spread of data, while the eigenvalue is the intensity of spread in a particular direction or of that respective eigenvector. Given the weighted correlation matrix $\mathbf {A}$ of network *G*, it is wise to choose the largest eigenvalue, $\lambda _{\text {max}}$, in the absolute value of matrix. By virtue of the Perron–Frobenius theorem [41], this choice implies that if the graph is strongly connected, then the eigenvector solution $\vec {c}$ is both unique and positive.

### EEG microstate analysis

For microstate analysis, we follow the standard steps in microstate segmentation presented in [44]. For this purpose, the EEG data at different bands were imported to MATLAB (vR2016a) using the EEGLAB toolbox (v14.1.2) [45, 46]. First, we need to calculate the global field power (GFP) at each data point which represents the magnitude of the field strength at each moment in time. The GFP at each data point is equal to the root of the mean of the squared potential differences at all *N* electrodes, i.e., $V_i(t)$, from the mean of instantaneous potentials across electrodes, i.e., $\overline{V_i}(t)$, equivalently, the standard deviation across all electrodes of the EEG for the *i*th data point [47]. Formally,

16 $$\begin{aligned} \text {GFP}(t)=\sqrt{\frac{\sum _{i=1}^{n}(V_i(t)-\overline{V_i}(t))^{2}}{n}}. \end{aligned}$$

**Fig. 2:**
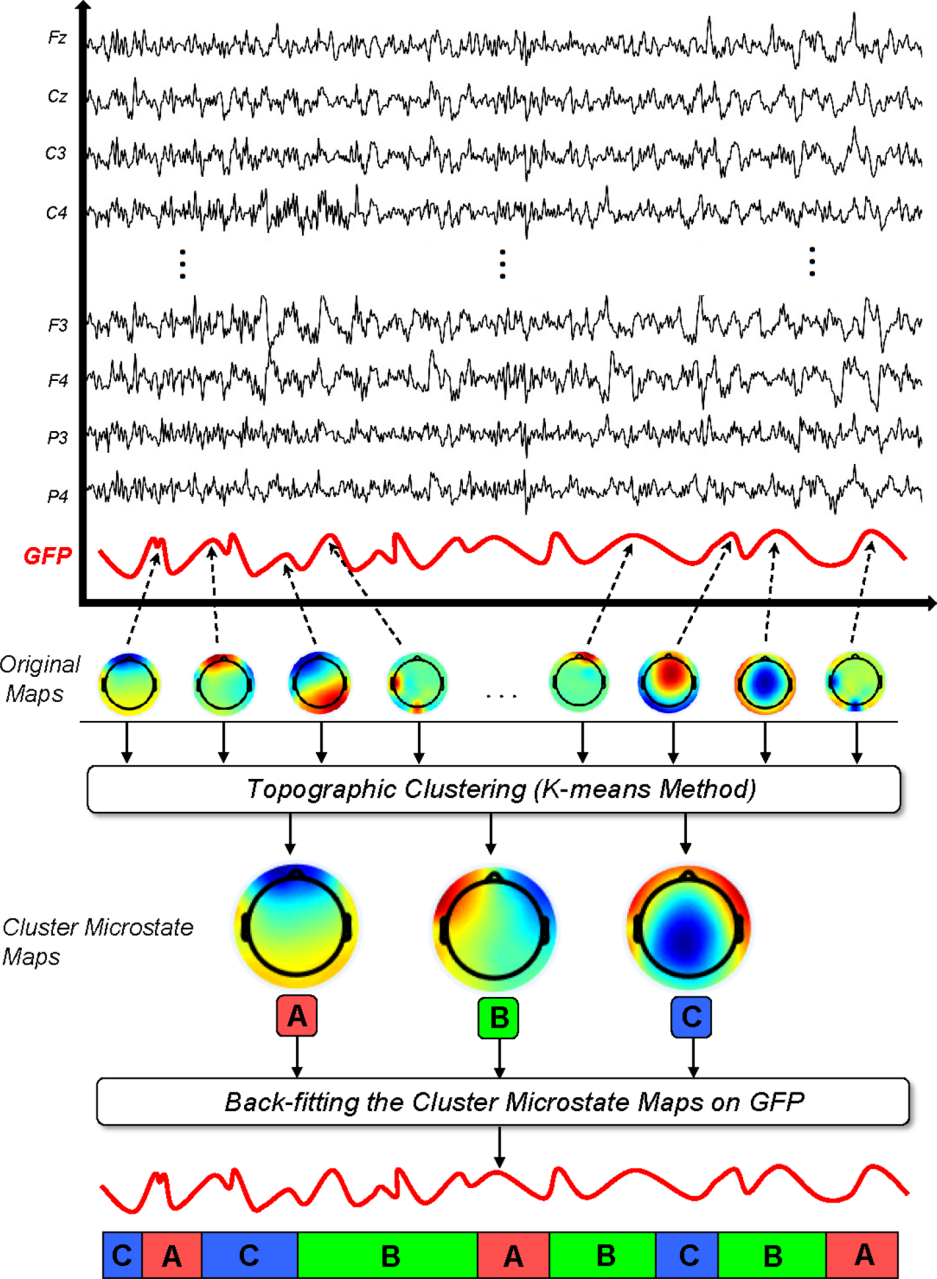
Schematic flowchart of the EEG microstate analysis. Each EEG datum is used to calculate the GFP curve at each data point. The electric potentials of all electrodes at moments of local maxima of the GFP curve are plotted to generate topographic original maps. The original maps are submitted to a clustering algorithm, which groups the submitted maps into a small set of clusters (here: 3) based on topographic similarity, and optimal number of cluster microstate maps is generated for each subject. Finally, the cluster maps are back-fitted to the GFP curve and each data point is labeled with the cluster map that they best correlated to. Therefore, the multichannel EEG recording is now described as a series of alternating microstates

Topographies that occur at local peaks of the GFP(*t*) curve represent instants of greatest field strength and highest SNR. Since the field topography remains essentially stable between two peaks of the GFP(*t*) curve and changes during the troughs, the topographies at GFP(*t*) maxima are representative of topographies at surrounding data points in time [18, 48]. Thus, representation of the EEG data as a set of topographies at local GFP(*t*) maxima is a valid data reduction method. Therefore for each subject, the topographies at local GFP(*t*) peaks are extracted. These topographies are called the original maps and which are submitted to a clustering algorithm, such as K-means, to obtain the desired number of cluster maps with the goal of maximizing the similarity between the EEG samples and the prototypes of the microstates they are assigned to. A schematic overview of the microstate analysis is shown in Fig. 2.

In this work we aim to compare the cluster maps of two different groups (i.e., epilepsy and PNES patient groups) and then identify patients using a machine learning technique. Each subject may result in different number of microstate cluster maps. Therefore, it would be quite complicated to compare the temporal characteristics of microstates' maps between the two groups. Hence, it would be ideal to have a set of global cluster maps that represent the recordings of all subjects in both group and then fit these common maps to the individual data for further investigations. Therefore, we apply a data aggregation scheme for each group, as 5000 original maps at GFP(*t*) maxima of each subject, with the minimum peak distance of 20 µs, are extracted and concatenated to create a new series of topographic original maps. This aggregated series explain variance in both of our datasets, consisting of 100 subjects including 50 epilepsy and 50 PNES. As the next step, the aggregated series is submitted to the modified K-means clustering algorithm to obtain the global microstate cluster maps.

#### Effective number of cluster maps

Finding the optimum number of cluster maps is crucial for capturing the informative features of the data and avoids over/under-fitting. Selecting the number of cluster microstates is not a straightforward choice to make [21, 49, 50]. In this paper, we apply cross-validation criterion [51] as a measure of fit for selecting the effective number of microstates, because this measure is polarity-invariant as it is assumed in the segmentation of spontaneous EEG data.

The cross-validation criterion (CV) [51] optimizes the ratio between the global explained variance and the degrees of freedom for a given set of cluster maps. This measure is related to the residual noise, $\epsilon$, and the goal is therefore to obtain a low value of CV.

17 $$\begin{aligned} \text {CV}=\hat{\sigma }^2 \cdot \left( \frac{C-1}{C-K-1}\right) ^2 \end{aligned}$$

where *C* is number of EEG channels, *K* number of microstate clusters and $\hat{\sigma }^2$ an estimator of the variance of the residual noise calculated as

18 $$\begin{aligned} \hat{\sigma }^2=\frac{\sum _n^N \mathbf{x} _n^T \mathbf{x} _n - (\mathbf{a} _{l_n}^T \mathbf{x} _n)^2 }{N(C-1)} \end{aligned}$$

where *N* is number of time samples, $\mathbf{x} _n$ is the *n*th time sample of the recorded EEG, $\mathbf{a} _{l_n}$ signifies the topographical map assigned to *n*th EEG sample and $l_n$ is the microstate label of the *n*-th EEG sample. Practically, the CV criterion pointing to the best clustering solution at its smallest value.

**Fig. 3:**
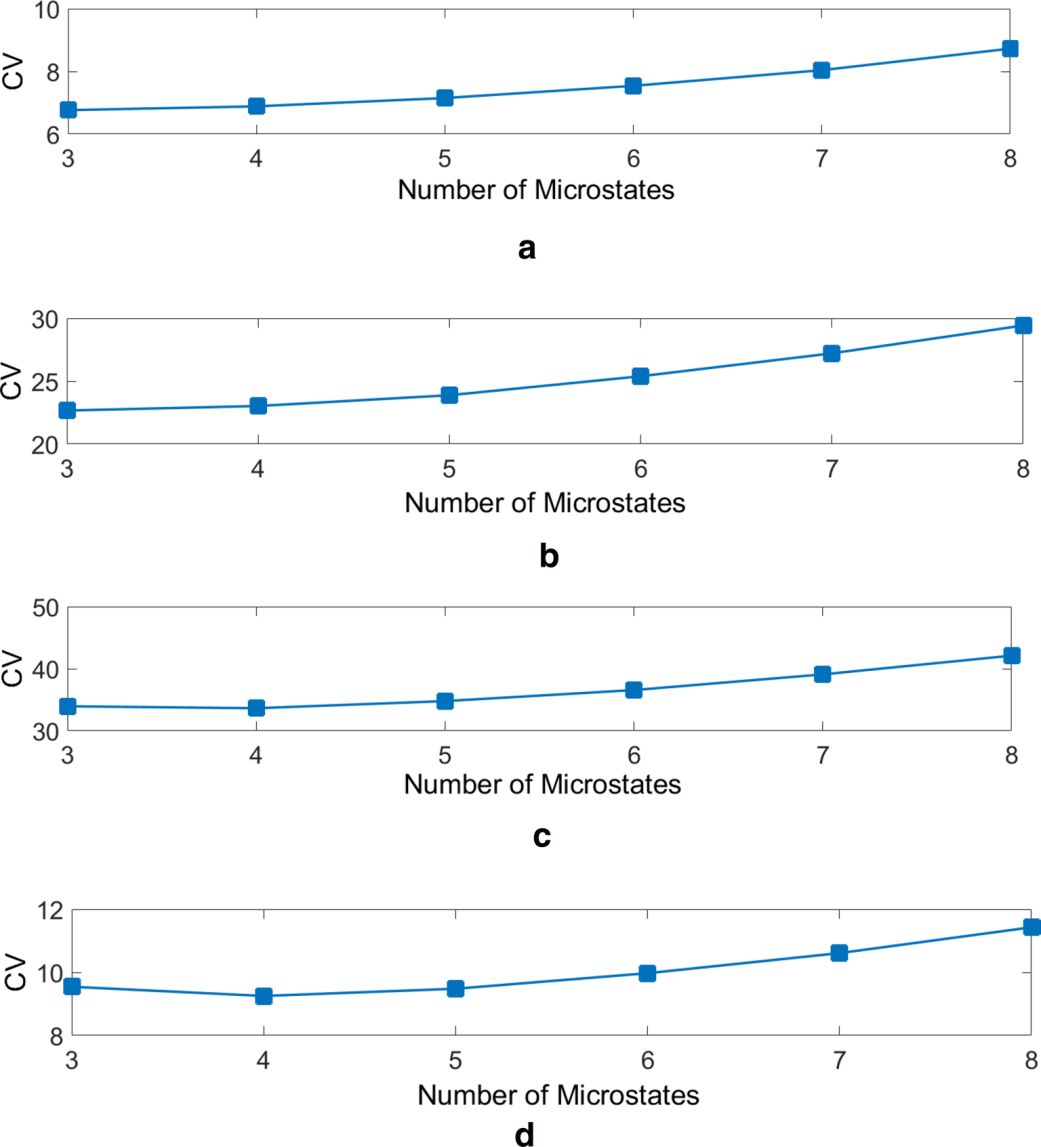
The CV measure of fit plotted for **a** alpha-band, **b** beta-band, **c** delta-band and **d** theta-band

**Fig. 4:**
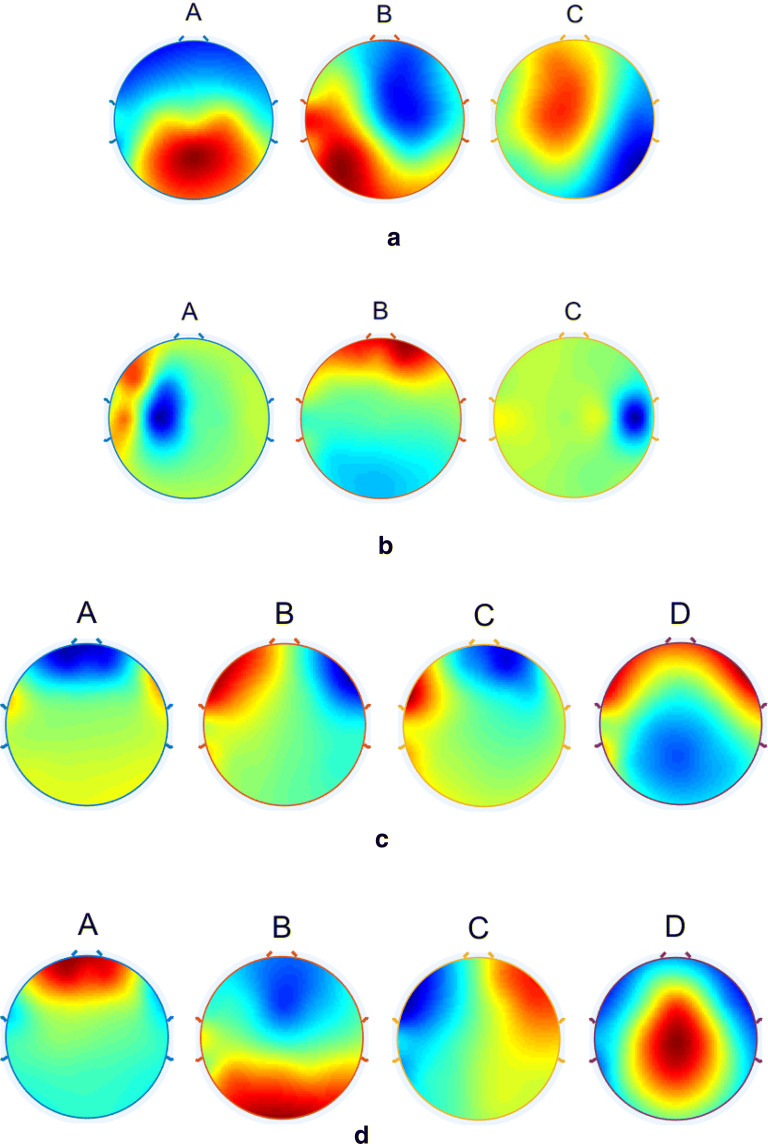
The topographies of the selected global microstate classes retrieved from the clustering algorithm for **a** alpha-band, **b** beta-band, **c** delta-band and **d** theta-band

The decision for the right number of clusters obviously reflects a trade-off between the goodness of fit and the complexity a high number of microstates brings to the segmentation. Hence according to the CV and GEV plots (see Fig. 3, the optimum numbers of global cluster maps are 3, for alpha and beta-bands, and 4 for delta and theta-bands. The topographies of the global cluster maps are shown in Fig. 4.

#### Back-fitting microstates maps to EEG

Once the global cluster maps have been determined, they are fitted-back to each individual subject’s EEG and its corresponding GFP(*t*) data to define the microstates and extract different features. Back-fitting procedure assigns microstate labels to EEG data point based on which cluster map they are most topographically similar with using the global map dissimilarity (GMD) measure. The GMD is a distance measure that is invariant to the strength of the signal and instead only looks at how similar the topographical maps look. For two EEG samples, $\mathbf{x} _n$ and $\mathbf{x} _n\prime$, GMD is calculated as

19 $$\begin{aligned} \text {GMD}=\frac{|| \frac{\mathbf{x }_n}{\mathbf{GFP }_n} - \frac{\mathbf{x }_n\prime }{\mathbf{GFP }_n\prime } ||}{\sqrt{C}}. \end{aligned}$$

By normalizing with GFP, two EEG samples that belong to the same microstate, but have different strength, will achieve a low GMD distance.

**Fig. 5:**
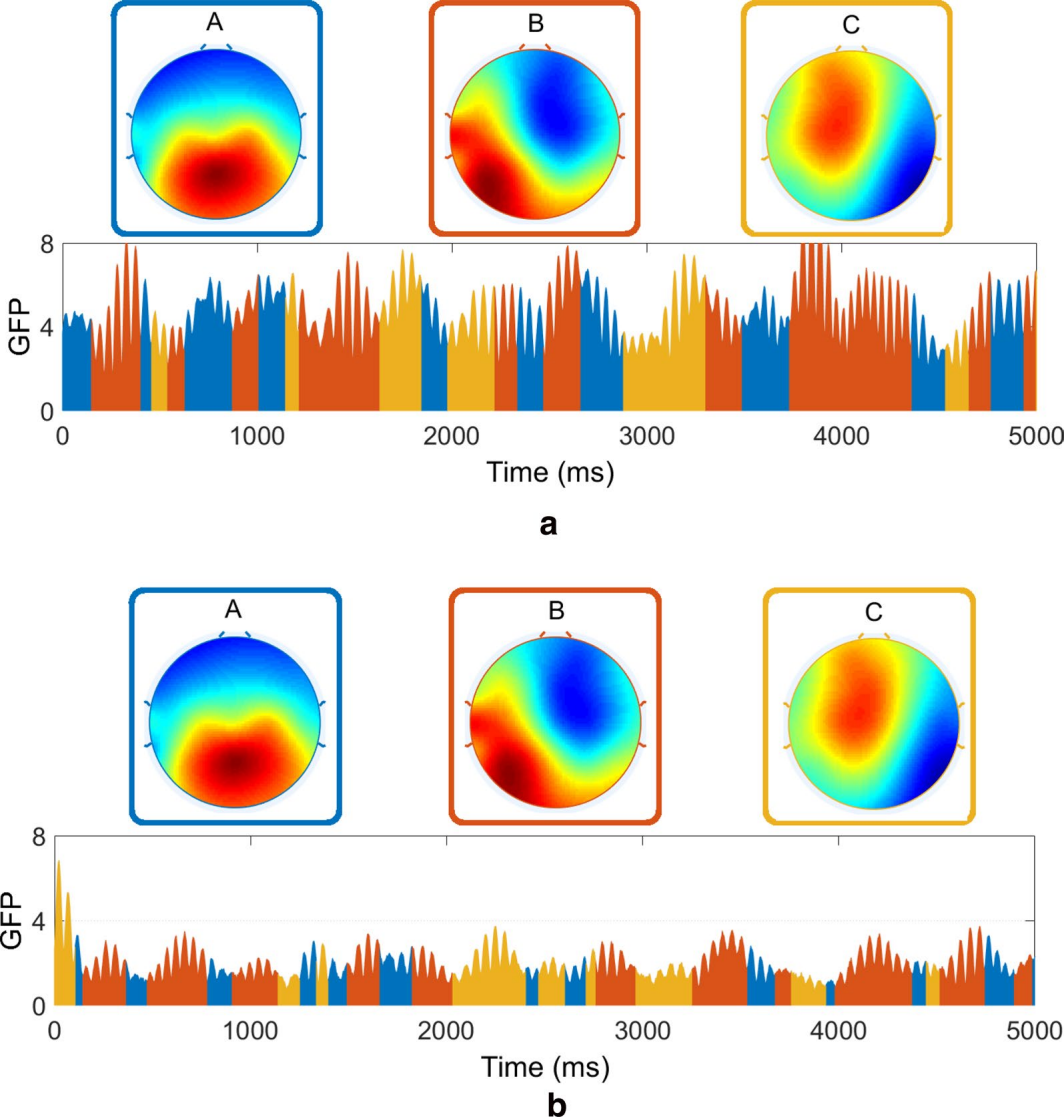
The global cluster maps are back-fitted to the GFP curve of **a** a subject with epilepsy and **b** a subject with PNES at beta-band. Each data point is labeled with the cluster map based on the maximal spatial correlation with the global template. The time period that each of the cluster maps covered is shown by color bars

Hence, the obtained global cluster maps are fitted backward to the original data calculating the spatial correlation between each template and the topography at each time instant corresponding to the maximum value of GFP. Such a procedure allows to represent the EEG time series in terms of sequence of microstates and to extrapolate variables of interest. Figure 5 shows an epoch of EEG data of two different subjects as a function of global cluster microstates at different EEG bands. It is worth mentioning that the unwanted noise in EEG recording can appear as a short microstate segments after the back-fitting procedure. To eliminate this issue, the small maps rejection algorithm is implemented to temporally smooth the microstates after the back-fitting. For this purpose, we introduce a threshold (here: 30 µs) which defines the minimum duration for the microstate segments to last. Hence, the label of each microstate segment with duration lower than the threshold changes to the next most likely microstate cluster map as measured by the GMD measure.

#### EEG microstate features

The basic temporal dynamics of microstates are described by occurrence (*k*), duration (*k*), and coverage (*k*). Occurrence (*k*) reflects the average number of times per second a microstate is dominant, the Duration (*k*) is defined as the average duration of a given microstate (in milliseconds), and the Coverage (*k*) reflects the fraction of time a given microstate is active. These features are inputted to various selected classifiers, independently.

### Training/test set split

As introduced in Sect. 2.1, there are totally 5 epilepsy and 5 PNES patients and each patient has 50 IED-free epochs. To augment the data, we transfer these 10 patients into 100 subjects including 50 Epilepsy and 50 PNES epochs. I.e., we transfer each patient into 10 subjects by diving the epochs with a fixed duration.

To avoid overfitting, we conduct classification experiments using cross-validation. However, the data are augmented from limited number of patients so it requires a specific data split to prevent the classifier learning the patterns of each patient. For this purpose, we randomly select 1 Epilepsy patient and 1 PNES patient where each patient includes 10 epochs with the same label (totally 20 subjects) as the test set. The rest 4 Epilepsy patients and 4 PNES patients (totally 80 subjects) are used as the training set. Therefore, there are totally $5\times 5=25$ pairs of cross-validation experiments since we have 5 epilepsy and 5 PNES patients. The results reported in this paper will be the average of these 25 pairs.

### Features classification

In machine learning and statistics, classification is a supervised learning approach in which the computer program learns from the data input with labels given to it and then uses this learning to classify new observation. In this work, we apply various well-known classification techniques, including k-Nearest-Neighbors [52], Decision Tree [53], Neural Network [54], Random Forest [55], Naive Bayes [56], Support Vector Machine with linear and radial kernels (SVM-Linear, SVM-RBF) [57] and Gradient Boosting [58], for classification of subjects. The specific kind of function being learned and the assumptions built into it are what distinguish among the various types of classifiers.

The usual model performance measures for evaluating a classification model are precision (or positive predictive value), recall (or sensitivity, true positive rate), accuracy and specificity (or true negative rate). Precision is calculated as the number of correct positive predictions divided by the total number of positive predictions. Recall is calculated as the number of correct positive predictions divided by the total number of positives. In other words, recall is the number of correct positives divided by the number of correct positives plus the number of false-negatives. True-positives are data point classified as positive by the model that actually are positive (meaning they are correct), and false-negatives are data points the model identifies as negative that actually are positive (incorrect). Recall gives us information about performance of the model on false-negatives, while precision gives us information of the model’s performance of false-positives. Based on what is predicted, precision or recall might be more critical for a model. Accuracy is the number of correct predictions made by the model by the total number of records. The best accuracy is 100$\%$ indicating that all the predictions are correct. Specificity is calculated as the number of correct negative predictions divided by the total number of negatives.

The receiver operating characteristic (ROC) curve is a plot of specificity in the $\mathbf{x}$ axis and recall in the $\mathbf{y}$ axis. Hence, the ROC curve is a plot of the false-positive rate ($\mathbf{x}$-axis) versus the true-positive rate ($\mathbf{y}$-axis) for a number of different subjects threshold values between 0.0 and 1.0. Area under the ROC curve is a measure of model performance. The area under the curve (AUC) of a random classifier is 50$\%$ and that of a perfect classifier is 100$\%$. For practical situations, an AUC of over 70$\%$ is desirable [59].

## Results

In this section, the classification results of epilepsy and PNES, in the absence of an interictal discharge, from real multichannel EEG data are presented based on the EEG signal, functional network and EEG microstate features.

### EEG features’ classification

In this section, the classification of EEG signal features which were extracted from each single channels is presented. Table 1 shows the performance of the selected EEG signal features in the classification task using different classifiers. Here, the results for two evaluation metrics, i.e., precision and recall, at different EEG frequency sub-bands are presented. It can be seen that different sub-bands have different performance w.r.t selected signal features. Conclusions for each individual sub-band are as following:

- In alpha-band, the spectral entropy performs best among the features and the SVM classifier (with linear and RBF kernels) performs best among the classification techniques. The Renyi entropy is the second best feature in the classification tasks. Besides, features such as Higuchi fractal dimension are the worst feature to distinguish subjects because it only achieves about 50$\%$ precision.
- In beta-band, all features except Higuchi fractal dimension achieve acceptable classification results. Similar to alpha-band, the SVM (with linear and RBF kernels) performs best among all classifiers, and the Higuchi fractal dimension is the worst feature to distinguish subjects.
- In delta-band, Katz Fractal dimension and signal energy perform relatively better than other features. Entropy-based features, including Shanon, spectral and Renyi, did not perform well in classifying subjects. Similarly, the SVM (with linear and RBF kernels) performs best in most features.
- In theta-band, Katz fractal dimension performs the best among all features. But other features achieve poor performance as the precision is around 50$\%$. Similarly, the SVM (with linear and RBF kernels) performs best in most features.
- In gamma-band, almost all features obtain poor performance except signal energy. It indicates that gamma-band may not be a very effective band for classifying the subjects in experiments.

**Table 1:** Calculated classification precision and recall using various classification techniques at different frequency bands

	Higuchi FD	Katz FD	Energy	Shanon entropy	Spectral entropy	Renyi entropy
Precision, Recall at alpha-band						
SVM (Linear)	0.545, 0.438	0.597, 0.446	0.624, 0.463	0.655, 0.505	0.741, 0.639	0.713, 0.603
SVM (RBF)	0.422, 0.367	0.641, 0.483	0.578, 0.424	0.529, 0.393	0.742, 0.641	0.669, 0.559
Gradient Boosting	0.552, 0.444	0.582, 0.480	0.566, 0.474	0.513, 0.428	0.720, 0.621	0.692, 0.601
Decision Tree	0.514, 0.394	0.697, 0.616	0.595, 0.512	0.575, 0.462	0.616, 0.495	0.667, 0.568
Random Forest	0.495, 0.406	0.602, 0.493	0.524, 0.425	0.584, 0.438	0.693, 0.598	0.665, 0.560
Precision, Recall at beta-band						
SVM (Linear)	0.601, 0.468	0.789, 0.677	0.755, 0.619	0.761, 0.652	0.743, 0.656	0.736, 0.651
SVM (RBF)	0.606, 0.476	0.754, 0.644	0.742, 0.597	0.730, 0.605	0.745, 0.651	0.774, 0.696
Gradient Boosting	0.527, 0.388	0.695, 0.513	0.646, 0.497	0.619, 0.474	0.716, 0.590	0.748, 0.657
Decision Tree	0.613, 0.482	0.695, 0.580	0.620, 0.483	0.556, 0.454	0.716, 0.617	0.706, 0.612
Random Forest	0.537, 0.433	0.727, 0.587	0.596, 0.474	0.685, 0.528	0.737, 0.637	0.724, 0.627
Precision, Recall at delta-band						
SVM (Linear)	0.633, 0.539	0.703, 0.549	0.686,0.539	0.510,0.342	0.542, 0.412	0.557, 0.452
SVM (RBF)	0.569, 0.482	0.665, 0.505	0.683, 0.543	0.581, 0.432	0.538, 0.426	0.479, 0.371
Gradient Boosting	0.622, 0.519	0.501, 0.345	0.573, 0.432	0.534, 0.380	0.561, 0.477	0.555, 0.431
Decision Tree	0.636, 0.539	0.553, 0.423	0.592, 0.485	0.475, 0.384	0.585, 0.617	0.552, 0.418
Random Forest	0.558, 0.468	0.583, 0.468	0.550, 0.457	0.503, 0.417	0.515, 0.414	0.573, 0.465
Precision, Recall at theta-band						
SVM (Linear)	0.633, 0.539	0.703, 0.549	0.686, 0.539	0.510, 0.342	0.542, 0.412	0.557, 0.452
SVM (RBF)	0.569, 0.482	0.665, 0.505	0.683, 0.543	0.581, 0.432	0.538, 0.426	0.479, 0.371
Gradient Boosting	0.622, 0.519	0.501, 0.345	0.573, 0.431	0.534, 0.380	0.561, 0.477	0.555, 0.431
Decision Tree	0.636, 0.539	0.553, 0.423	0.592, 0.485	0.475, 0.384	0.585, 0.617	0.552, 0.418
Random Forest	0.558, 0.468	0.583, 0.468	0.550, 0.457	0.503, 0.417	0.515, 0.414	0.573, 0.465
Precision, Recall at gamma-band						
SVM (Linear)	0.331, 0.251	0.583, 0.470	0.724, 0.552	0.539, 0.432	0.539, 0.340	0.537, 0.390
SVM (RBF)	0.409, 0.324	0.632, 0.481	0.686, 0.501	0.642, 0.501	0.501, 0.346	0.501, 0.389
Gradient Boosting	0.382, 0.317	0.743, 0.651	0.556, 0.442	0.488, 0.406	0.488, 0.368	0.525, 0.403
Decision Tree	0.468, 0.369	0.761, 0.650	0.550, 0.481	0.559, 0.478	0.505, 0.361	0.548, 0.420
Random Forest	0.381, 0.353	0.632, 0.490	0.613, 0.537	0.578, 0.462	0.524, 0.415	0.523, 0.419

**Fig. 6:**
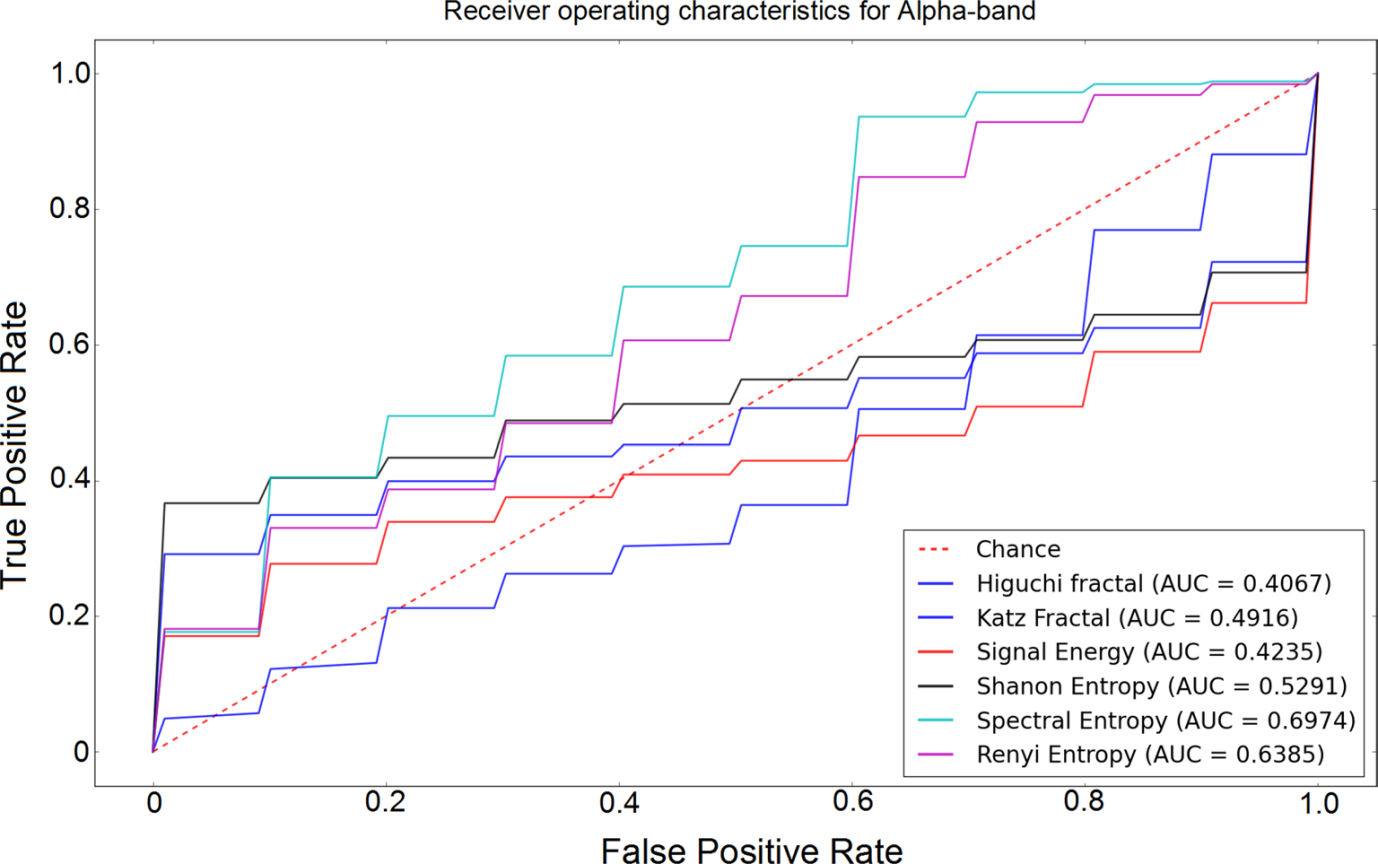
ROC analysis of the classification method using various EEG signal features in alpha-band

**Fig. 7:**
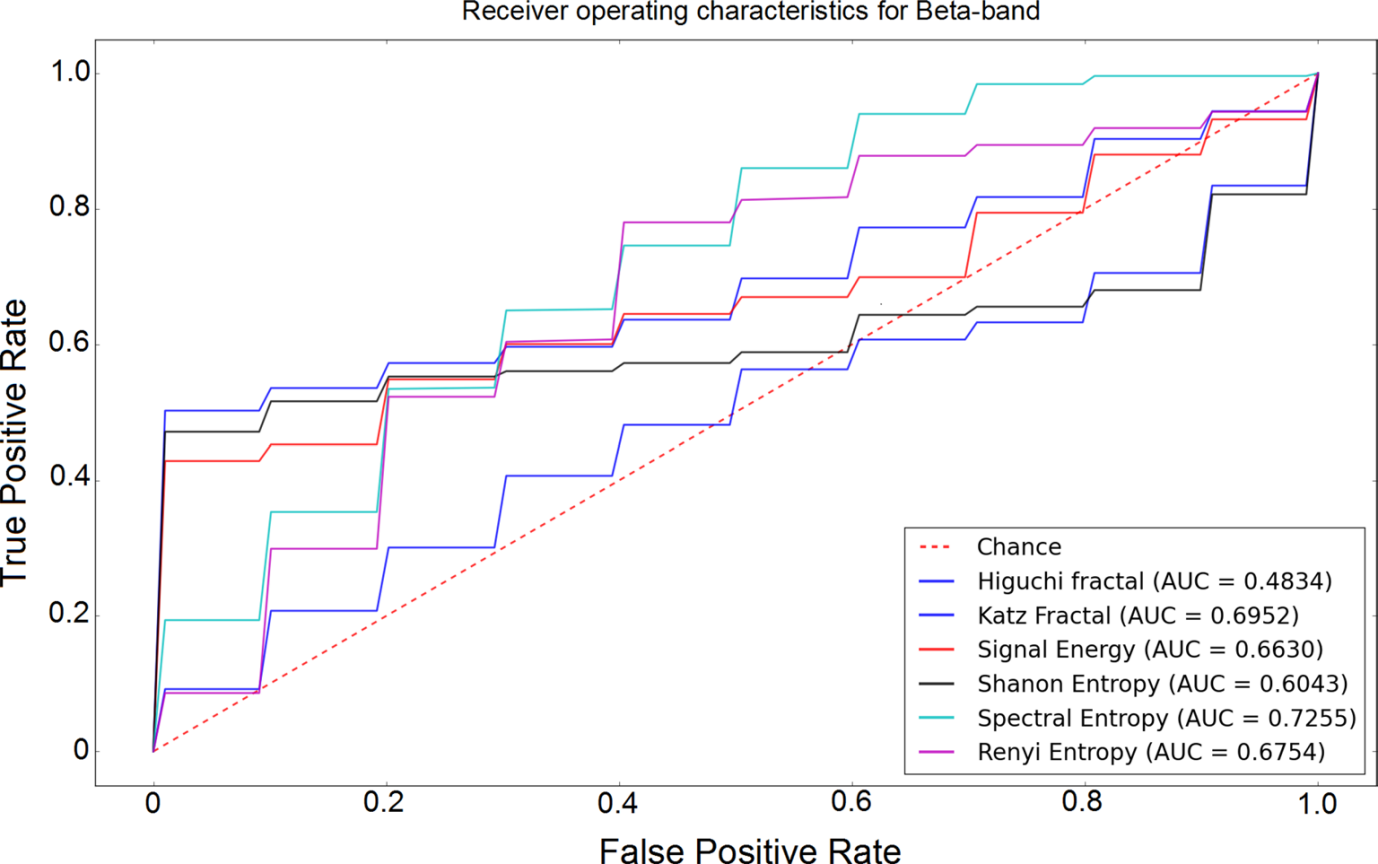
ROC analysis of the classification method using various EEG signal features in beta-band

**Fig. 8:**
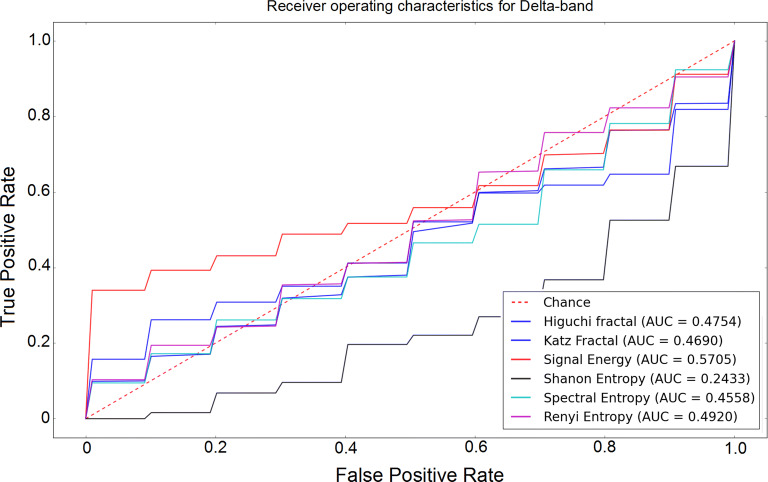
ROC analysis of the classification method using various EEG signal features in delta-band

**Fig. 9:**
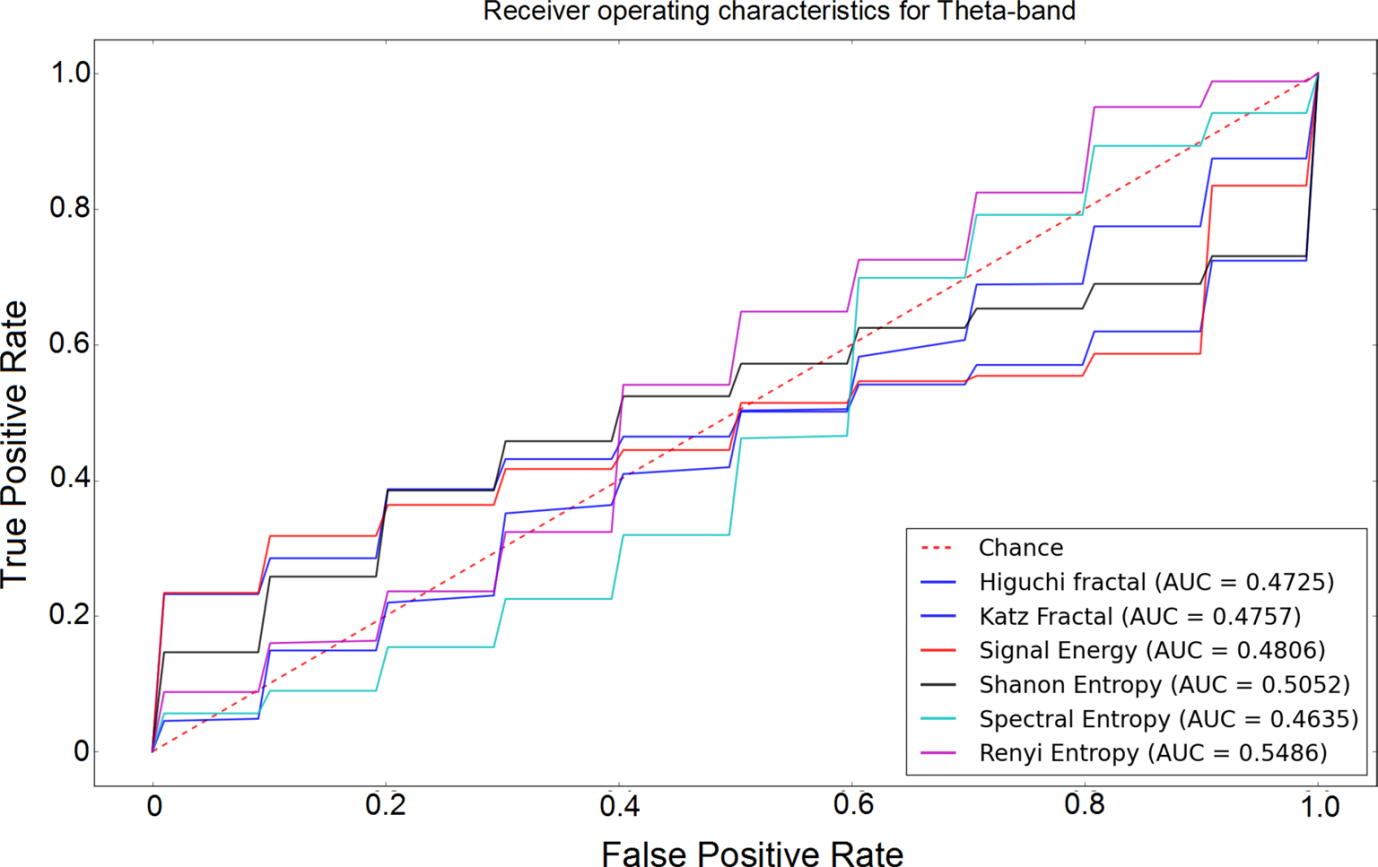
ROC analysis of the classification method using various EEG signal features in theta-band

**Fig. 10:**
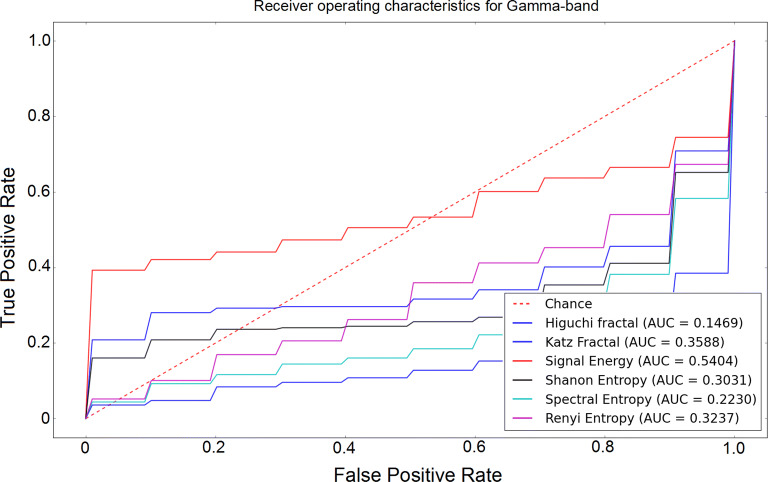
ROC analysis of the classification method using various EEG signal features in gamma-band

The receiver operating characteristic (ROC) curves for different sub-bands are shown in Figs. 6, 7, 8, 9 and 10. Considering the area under the curves (AUC) we can conclude that the classifiers that use features extracted from the beta-band perform best in classifying subjects as the AUC is the beta-band that is higher than other sub-bands for all selected features. Also, it is observed that the gamma-band performs worst using different features. Furthermore, the alpha-band performs relatively good but still much worse than beta-band. The delta-band and the theta-band are similar to random guess.

We also considered a combination of all selected features as the input for the classifiers. The results are presented in Table 2. It can be seen that by combining all the selected features, the classification precision and recall become larger for all sub-bands.

**Table 2:** Classification precision and recall calculated by the classifiers using combination of all features at different frequency bands

	Alpha-band	Beta-band	Delta-band	Theta-band	Gamma-band
Precision, Recall					
SVM (Linear)	0.615, 0.528	0.613, 0.555	0.674, 0.523	0.685, 0.615	0.751, 0.664
SVM (RBF)	0.622, 0.547	0.741, 0.593	0.618, 0.554	0.613, 0.444	0.783, 0.688
Gradient Boosting	0.577, 0.513	0.611, 0.512	0.667, 0.499	0.569, 0.466	0.781, 0.688
Decision Tree	0.574, 0.447	0.713, 0.716	0.685, 0.532	0.601, 0.514	0.688, 0.577
Random Forest	0.583, 0.502	0.694, 0.532	0.587, 0.495	0.604, 0.488	0.766, 0.644

### Network features classification

By applying the horizontal visibility graph algorithm, the synchronizations among all pairs of EEGs are calculated. Then, the correlation matrices and corresponding functional brain networks are constructed to extract selected network measures, i.e., clustering coefficient, strength, betweenness centrality, eigenvector centrality and largest eigenvalue (see Sect. 2.2). At first, the classification techniques were applied on each network measure independently. However, the classification results were poor. Hence, a combination of all selected features was considered as the input for the classifiers.

The precision, recall and accuracy of the classification methods with the best performances for the combination of all the networks’ features at different EEG bands are presented in Table 3. From these results, we can say that the functional network features are not strong discriminative features to be used for the classification of the epileptic seizure and PNES. However from the results, we can conclude that functional network features are robust to the classification task, i.e., different bands perform similarly in classification precision/recall. Among different bands, gamma-band performs best while theta-band performs worst. Also, among different applied classifiers, the SVM either with linear or with RBF kernel performs best for all EEG bands. The only exception is delta-band where Random Forest classifier performs best. Note that for the gamma-band the results of the SVM (RBF) are about 5$\%$ less than the results of the Random Forest technique.

**Table 3:** Classification precision, recall and accuracy calculated by the classifiers with the best performance for different EEG bands

EEG-band	Model	Precision	Recall	Accuracy
Alpha	SVM (RBF)	0.6906	0.592	0.592
Beta	SVM (Linear)	0.6825	0.638	0.638
Delta	Random Forest	0.6843	0.584	0.588
Theta	SVM (RBF)	0.6492	0.534	0.534
Gamma	SVM (Linear)	0.7001	0.554	0.554

### Microstate features classification

These microstate features are inputted to various selected classifiers, independently. The classification precision, recall and accuracy are presented in Tables 4, 5 and 6. Also, Table 7 presents the classification results when all three features are considered as inputs for classifiers. From these results it can be seen that the microstate analysis in beta-band leads to more accurate results compared to other EEG bands. Also, the kNN classifier is a superior technique for doing classification. The only exception is when coverage (*k*) is the classification input, where Random Forest classifier performs slightly better than the kNN model. Furthermore, it is observed that the occurrence (*k*) is the weakest discriminative feature as it results in overall accuracy of 68.8$\%$ with 72.8$\%$ precision and 68.9$\%$ recall, whereas duration (*k*), coverage (*k*) and combination of all features mostly result in accuracy, precision and recall higher than 80$\%$.

**Table 4:** Classification precision, recall and accuracy calculated by selected classifiers at different EEG data bands when occurrence (*k*) is considered as the discriminative (or input) feature for classification

	Alpha	Beta	Delta	Theta
Accuracy, Precision, Recall				
Random Forest	0.522, 0.522, 0.522	0.702, 0.763, 0.702	0.480, 0.480, 0.480	0.534, 0.498, 0.534
SVM (Linear)	0.516, 0.519, 0.516	0.766, 0.808, 0.766	0.326, 0.316, 0.326	0.506, 0.512, 0.506
SVM (RBF)	0.508, 0.519, 0.508	0.724, 0.782, 0.724	0.424, 0.416, 0.424	0.576, 0.573, 0.576
Decision Tree	0.480, 0.477, 0.480	0.666, 0.683, 0.666	0.588, 0.601, 0.588	0.558, 0.555, 0.558
kNN	0.534, 0.535, 0.534	0.688, 0.728, 0.689	0.602, 0.615, 0.602	0.646, 0.655, 0.646
Gradient Boost	0.526, 0.524, 0.526	0.688, 0.721, 0.688	0.614, 0.623, 0.614	0.620, 0.632, 0.620

**Table 5:** Classification precision, recall and accuracy calculated by selected classifiers at different EEG data bands when duration (*k*) is considered as the discriminative (or input) feature for classification

	Alpha	Beta	Delta	Theta
Accuracy, Precision, Recall				
Random Forest	0.552, 0.558, 0.552	0.694, 0.757, 0.694	0.506, 0.5064, 0.506	0.530, 0.539, 0.530
SVM (Linear)	0.430, 0.2539, 0.430	0.522, 0.431, 0.522	0.478, 0.336, 0.478	0.512, 0.432, 0.512
SVM (RBF)	0.498, 0.429, 0.498	0.502, 0.291, 0.502	0.514, 0.440, 0.514	0.498, 0.249, 0.498
Decision Tree	0.568, 0.5721, 0.568	0.730, 0.752, 0.730	0.544, 0.546, 0.544	0.604, 0.613, 0.604
kNN	0.576, 0.5838, 0.576	0.808, 0.839, 0.808	0.560, 0.584, 0.560	0.660, 0.668, 0.660
Gradient Boost	0.580, 0.587, 0.580	0.718, 0.751, 0.718	0.612, 0.618, 0.612	0.640, 0.653, 0.640

**Table 6:** Classification precision, recall and accuracy calculated by selected classifiers at different EEG data bands when coverage (*k*) is considered as the discriminative (or input) feature for classification

	Alpha	Beta	Delta	Theta
Accuracy, Precision, Recall				
Random Forest	0.478, 0.487, 0.478	0.794, 0.840, 0.794	0.508, 0.543, 0.508	0.556, 0.563, 0.556
SVM (Linear)	0.492, 0.487, 0.492	0.668, 0.708, 0.668	0.416, 0.410, 0.416	0.546, 0.553, 0.546
SVM (RBF)	0.474, 0.479, 0.474	0.656, 0.688, 0.656	0.396, 0.383, 0.396	0.442, 0.433, 0.442
Decision Tree	0.502, 0.498, 0.502	0.764, 0.783, 0.764	0.578, 0.586, 0.578	0.620, 0.631, 0.620
kNN	0.564, 0.571, 0.564	0.776, 0.814, 0.776	0.548, 0.551, 0.548	0.596, 0.612, 0.596
Gradient Boost	0.546, 0.549, 0.546	0.788, 0.826, 0.788	0.636, 0.652, 0.636	0.602, 0.614, 0.602

**Table 7:** Classification precision, recall and accuracy calculated by selected classifiers at different EEG data bands when the combination of occurrence (*k*), duration (*k*) and coverage (*k*) is considered as the discriminative (or input) feature for classification

	Alpha	Beta	Delta	Theta
Accuracy, Precision, Recall				
Random Forest	0.516, 0.518, 0.516	0.728, 0.792, 0.728	0.492, 0.504, 0.492	0.594, 0.609, 0.594
SVM (Linear)	0.432, 0.259, 0.432	0.604, 0.565, 0.604	0.464, 0.341, 0.464	0.480, 0.361, 0.480
SVM (RBF)	0.504, 0.531, 0.504	0.530, 0.528, 0.530	0.514, 0.440, 0.514	0.498, 0.249, 0.498
Decision Tree	0.570, 0.575, 0.570	0.716, 0.738, 0.716	0.584, 0.592, 0.584	0.582, 0.587, 0.582
kNN	0.576, 0.584, 0.576	0.808, 0.839, 0.808	0.566, 0.591, 0.566	0.662, 0.670, 0.662
Gradient Boost	0.572, 0.575, 0.572	0.808, 0.831, 0.808	0.650, 0.663, 0.650	0.634, 0.645, 0.634

To further evaluate the importance of the frequency bands, the so-called leave-one-out tests are performed. For this purpose, each microstate feature (i.e., occurrence (*k*), duration (*k*) and precision (*k*) and also combination of all three features) in all bands (i.e., alpha, beta, delta and theta) is inputted to classifiers independently to measure accuracy, precision and recall of the classification. The results of this test are shown under the header of All in Table 8. Then, we eliminate one of the frequency bands and do the classification again. The results are shown as All-alpha, All-beta, All-delta and All-theta in Table 8. The results show that the alpha, delta and theta-bands do not contain important data for microstate analysis as by eliminating them from the classification procedure, the accuracy, precision ad recall not only does not decrease significantly, but also become pronounced for some cases. However, the results for the beta frequency band are quite different. It can be seen that by eliminating the beta-band from the classification, the values of accuracy, precision and recall reduce significantly which highlight the importance of the beta-band in microstate analysis. This importance is confirmed by all selected classification techniques presented in this work.

**Table 8:** Calculated classification accuracy, precision and recall using all frequency bands (All), and excluding alpha-band (All-alpha), beta-band (All-beta), delta-band (All-delta) and theta-band (All-theta)

	All	All-alpha	All-beta	All-delta	All-theta
Accuracy, Precision, Recall using occurrence (*k*) as the discriminative (input) feature					
Random Forest	0.696, 0.732, 0.696	0.700, 0.738, 0.700	0.536, 0.537, 0.536	0.708, 0.741, 0.708	0.734, 0.784, 0.734
SVM (Linear)	0.732, 0.758, 0.732	0.734, 0.768, 0.734	0.396, 0.391, 0.396	0.724, 0.756, 0.724	0.700, 0.725, 0.700
SVM (RBF)	0.730, 0.774, 0.730	0.724, 0.772, 0.724	0.524, 0.535, 0.524	0.728, 0.772, 0.728	0.758, 0.793, 0.758
Decision Tree	0.646, 0.683, 0.646	0.680, 0.711, 0.680	0.602, 0.615, 0.602	0.656, 0.682, 0.656	0.680, 0.715, 0.680
kNN	0.794, 0.814, 0.794	0.822, 0.841, 0.822	0.694, 0.708, 0.694	0.762, 0.788, 0.762	0.802, 0.831, 0.802
Gradient Boost	0.692, 0.729, 0.692	0.696, 0.746, 0.696	0.654, 0.669, 0.654	0.688, 0.732, 0.688	0.698, 0.731, 0.698
Accuracy, Precision, Recall using duration (*k*) as the discriminative (input) feature					
Random Forest	0.686, 0.735, 0.686	0.684, 0.741, 0.684	0.538, 0.544, 0.538	0.674, 0.747, 0.674	0.670, 0.731, 0.670
SVM (Linear)	0.690, 0.740, 0.690	0.740, 0.806, 0.740	0.464, 0.401, 0.464	0.614, 0.668, 0.614	0.662, 0.733, 0.662
SVM (RBF)	0.614, 0.758, 0.614	0.590, 0.713, 0.590	0.598, 0.623, 0.598	0.694, 0.716, 0.694	0.578, 0.678, 0.578
Decision Tree	0.662, 0.685, 0.662	0.718, 0.743, 0.718	0.584, 0.587, 0.584	0.730, 0.758, 0.730	0.644, 0.668, 0.644
kNN	0.724, 0.746, 0.724	0.744, 0.773, 0.744	0.530, 0.541, 0.530	0.776, 0.820, 0.776	0.740, 0.765, 0.740
Gradient Boost	0.754, 0.796, 0.754	0.772, 0.810, 0.772	0.632, 0.636, 0.632	0.722, 0.767, 0.722	0.710, 0.743, 0.710
Accuracy, Precision, Recall using coverage (*k*) as the discriminative (input) feature					
Random Forest	0.730, 0.776, 0.730	0.734, 0.782, 0.734	0.560, 0.567, 0.560	0.710, 0.769, 0.710	0.754, 0.794, 0.754
SVM (Linear)	0.674, 0.702, 0.674	0.664, 0.696, 0.664	0.460, 0.439, 0.460	0.672, 0.707, 0.672	0.672, 0.704, 0.672
SVM (RBF)	0.618, 0.6631, 0.618	0.620, 0.666, 0.620	0.380, 0.354, 0.380	0.634, 0.681, 0.634	0.616, 0.656, 0.616
Decision Tree	0.698, 0.711, 0.698	0.738, 0.753, 0.738	0.682, 0.692, 0.682	0.678, 0.704, 0.678	0.628, 0.661, 0.628
kNN	0.776, 0.806, 0.776	0.764, 0.786, 0.764	0.634, 0.644, 0.634	0.778, 0.802, 0.778	0.738, 0.784, 0.738
Gradient Boost	0.762, 0.804, 0.762	0.782, 0.815, 0.782	0.696, 0.707, 0.696	0.758, 0.797, 0.758	0.748, 0.792, 0.748
Accuracy, Precision, Recall using combination of occurrence (*k*), duration (*k*) and coverage (*k*) as the discriminative (input) feature					
Random Forest	0.678, 0.738, 0.678	0.698, 0.764, 0.698	0.562, 0.567, 0.562	0.688, 0.757, 0.688	0.710, 0.779, 0.710
SVM (Linear)	0.712, 0.753, 0.712	0.734, 0.792, 0.734	0.514, 0.488, 0.514	0.612, 0.634, 0.612	0.656, 0.742, 0.656
SVM (RBF)	0.530, 0.337, 0.530	0.500, 0.250, 0.500	0.494, 0.308, 0.494	0.514, 0.393, 0.514	0.508, 0.268, 0.508
Decision Tree	0.692, 0.725, 0.692	0.698, 0.719, 0.698	0.612, 0.624, 0.612	0.660, 0.690, 0.660	0.646, 0.678, 0.646
kNN	0.724, 0.746, 0.724	0.744, 0.773, 0.744	0.530, 0.540, 0.530	0.776, 0.820, 0.776	0.740, 0.7652, 0.740
Gradient Boost	0.754, 0.792, 0.754	0.772, 0.805, 0.772	0.702, 0.712, 0.702	0.714, 0.763, 0.714	0.758, 0.786, 0.758

**Fig. 11:**
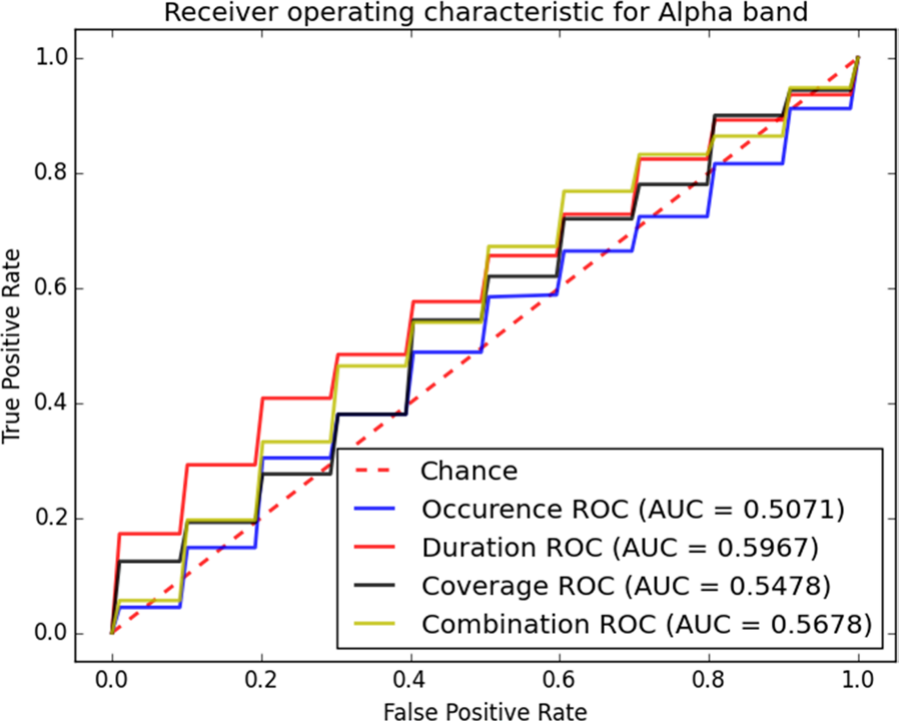
ROC analysis of the classification method using various microstate features in alpha-band

**Fig. 12:**
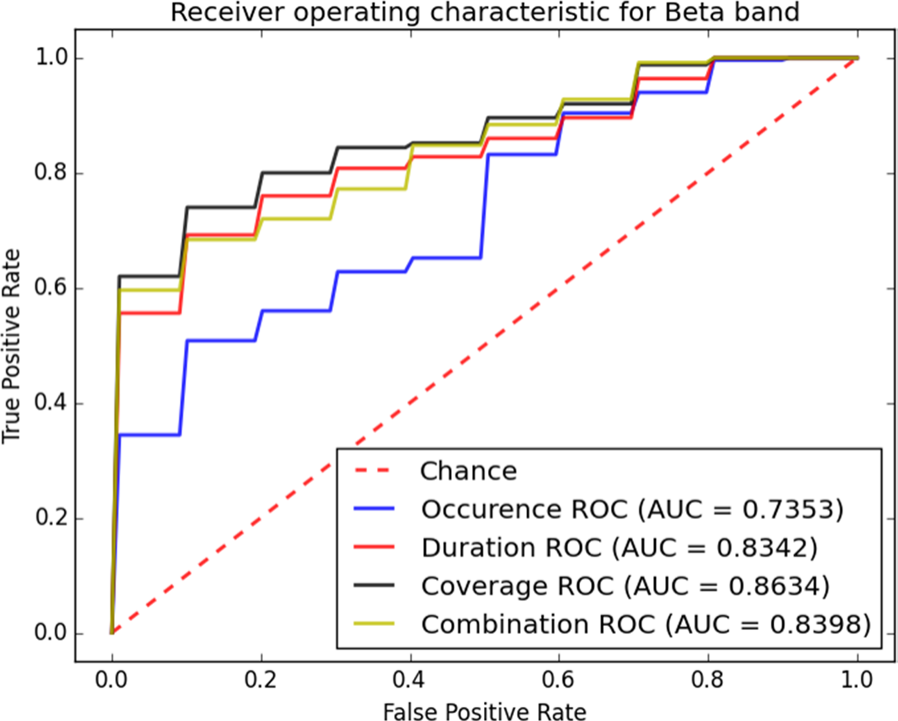
ROC analysis of the classification method using various microstate features in beta-band

**Fig. 13:**
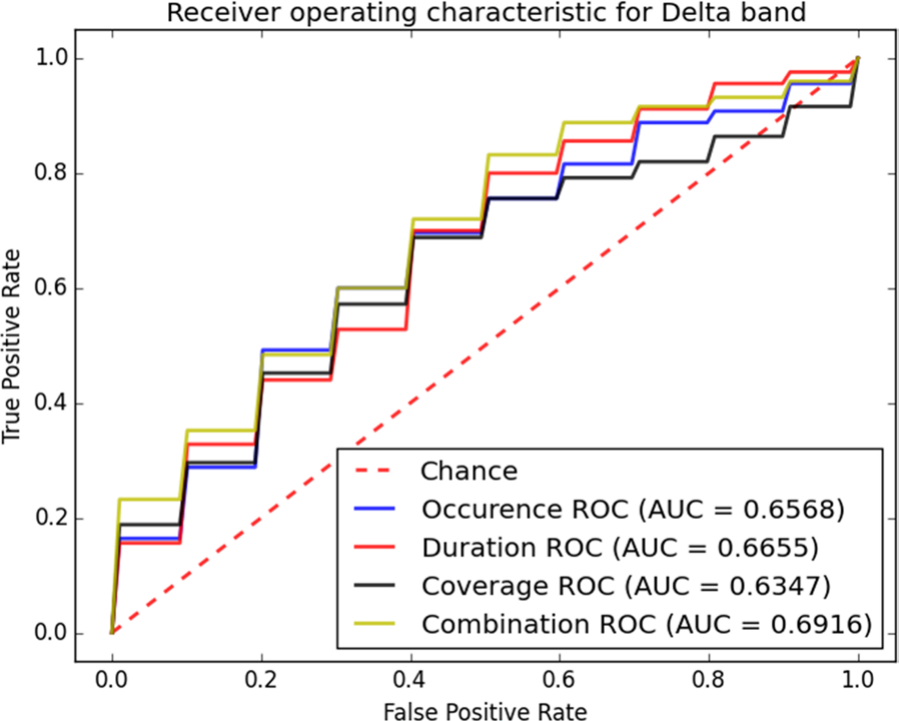
ROC analysis of the classification method using various microstate features in delta-band

**Fig. 14:**
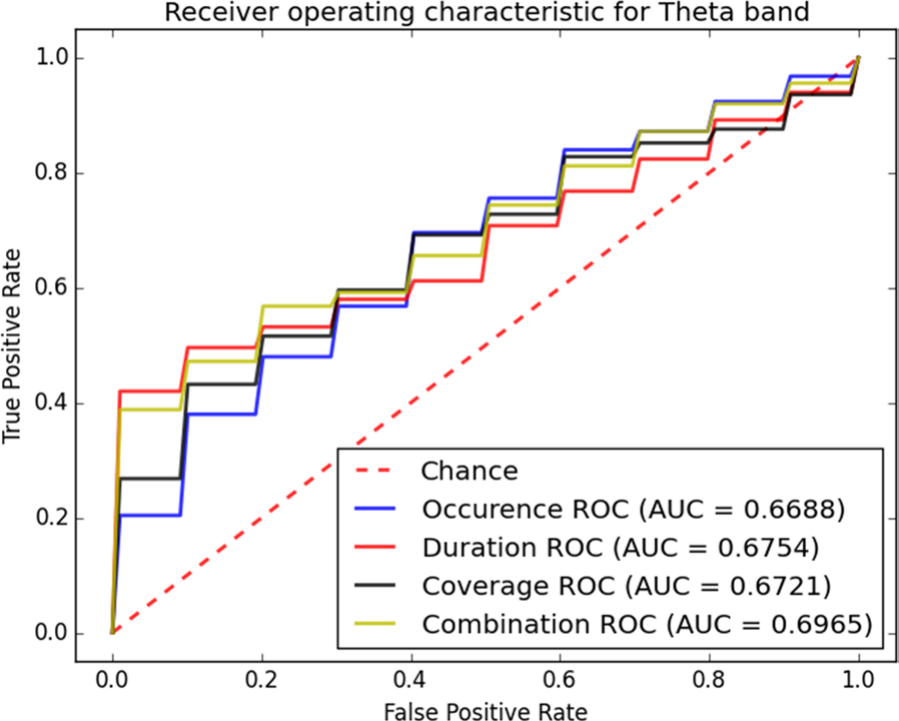
ROC analysis of the classification method using various microstate features in theta-band

The classification accuracy of the proposed system is also evaluated through receiver operating characteristic (ROC) curves for different microstate measures shown in Figs. 11, 12, 13 and 14. From these curves it can be seen that the area under the curve (AUC) of ROC in beta-band is larger for all microstate measures. This indicates that the beta-band is most accurate sub-band for our classification purpose. Furthermore, it is obvious from Fig. 12 that the coverage mainly results in larger AUC compared to other presented measures. The importance of the microstate features is presented in Table 9. The results show that by leaving out the coverage from the classification in beta-band, the accuracy, precision and recall of the classification reduce significantly compared to other measures. Hence, the coverage and beta-band are the most important features for classification of epileptic seizure and PNES using the microstate analysis.

**Table 9:** Importance of different microstate features

	Combination of all features	All without occurrence	All without duration	All without coverage
Accuracy, Precision, Recall				
Alpha-band	0.572, 0.575, 0.572	0.562, 0.566, 0.562	0.546, 0.546, 0.546	0.552, 0.557, 0.552
Beta-band	0.808, 0.851, 0.808	0.820, 0.863, 0.820	0.794, 0.836, 0.794	0.774, 0.811, 0.774
Delta-band	0.650, 0.663, 0.650	0.646, 0.658, 0.646	0.618, 0.625, 0.618	0.696, 0.714, 0.696
Theta-band	0.634, 0.645, 0.634	0.634, 0.639, 0.634	0.624, 0.635, 0.624	0.628, 0.639, 0.628

## Conclusion

In this paper, we investigated the EEG signal and functional brain network features for the automatic classification of epilepsy and PNES patients. An epileptic seizure is a transient occurrence of signs due to abnormal excessive or synchronous neuronal activity in the brain, where as PNES are events resembling an epileptic seizure, but without the characteristic electrical discharges associated with epileptic seizure. Hence, in the absence of the electrical discharge, the PNES is commonly misdiagnosed as an epileptic seizure. Generally, by performing a long-time EEG monitoring and recording the physicians can see if epileptiform discharges occur that aid in diagnosing the disorder. However, this monitoring is quite expensive and time-consuming. Hence, we aimed to effectively classify these two brain disorders in the absence of a seizure by analyzing various short-term EEG signal and network features using machine learning algorithms. All of our results showed that the beta-band is the most representative frequency sub-band for subject classification. Generally, the classification based on the EEG signal features and functional network features does not lead to classification with a strong performance even if various classification techniques are applied. The prediction accuracy was found to be around 80$\%$ when the classification was computed based on the microstate features extracted from the beta-bands.

## Data Availability

The data were made available by the UZ Gent hospital, Belgium only to Eindhoven University of Technology for performing experiments.
